# Boosting Psychological Well-Being through a Social Mindfulness-Based Intervention in the General Population

**DOI:** 10.3390/ijerph17228404

**Published:** 2020-11-13

**Authors:** Teresa Fazia, Francesco Bubbico, Gerardo Salvato, Giovanni Berzuini, Salvatore Bruno, Gabriella Bottini, Luisa Bernardinelli

**Affiliations:** 1 Department of Brain and Behavioral Sciences, University of Pavia, 27100 Pavia, Italy; francesco.bubbico01@universitadipavia.it (F.B.); gerardosalvato@gmail.com (G.S.); giomanuel_b@hotmail.com (G.B.); g.bottini@unipv.it (G.B.); luisa.bernardinelli@unipv.it (L.B.); 2 Cognitive Neuropsychology Centre, ASST “Grande Ospedale Metropolitano” Niguarda, 20162 Milano, Italy; 3 Istituto di Psicosintesi, 20124 Milano, Italy; salvab.counselor@gmail.com

**Keywords:** mindfulness-based meditation, psychological well-being, healthy subjects, public health

## Abstract

The benefits of mindfulness meditation among clinical and non-clinical populations have been largely reported in literature. Existing mindfulness-based programs are particularly useful in targeting specific populations while researchers have pointed out the possibility of developing programs adapted to the audience and the context. In this two-groups pre-post experimental design we developed a mindfulness-based social intervention program to target individuals from the general population. Here we present a two-groups pre-post experimental design to investigate its effectiveness on participants’ psychological functioning assessed by eight self-reported questionnaires (CORE-OM, FFMQ, SWLS, PANAS, PSS, SCS, WEMWBS, SHS) which encompass different domains of well-being, mindfulness and emotional functioning. Participants, recruited on voluntary basis, were randomly allocated to treated or passive control groups and were aware of group allocation. The intervention comprises a 12-week meditation training in a big group that represents the social aspect of meditation. Data were analysed via a linear mixed effect model and intention to treat. Statistically significant results were obtained for global score of CORE-OM (β = −0.20 [−0.30; −0.10], *p* = 0.0002), FFMQ (β = 0.20 [0.12; −0.28], *p* < 0.0001), SWLS (β = 1.43 [0.42; 2.45], *p* = 0.006), positive PANAS (β = 1.99 [0.95; 3.04], *p* = 0.0002), negative PANAS (β = −1.67 [−2.92; −0.43], *p* = 0.009), PSS (β = −2.98 [−4.25; −1.71], *p* < 0.0001), WEMWBS (β = 4.38 [2.93; 5.83], *p* < 0.0001) and SHS (β = 1.43 [0.42; 2.45], *p* = 0.006). Our intervention is causally associated with an improvement of the psychological functioning and hence can be considered as a preventive measure that may potentially reduce the risk of developing psychological problems and improve the subject’s general well-being. Given the voluntary recruitment, our inference only applies to those individuals who have decided to experience meditation as a way to well-being and not to a random person from the general population.

## 1. Introduction

Over the last decades, mindfulness-based programs (MPBs) have gained a lot of attention both in scientific research and clinical practice [1,2,3]. Being an easy-to-learn, economic and efficient technique, mindfulness training might be applied in different settings and nowadays it is integrated in many institutional environments like schools, prisons, workplaces [4]. As it produces physical, affective and cognitive benefits mindfulness-based intervention (MBI) may be exploited either as a treatment for symptoms of mental health disorders and other pathological conditions such as addiction, or just as a tool for increasing the quality of life, reducing stress and improving emotional functioning [5,6,7].

Furthermore, emerging evidence has shown that MBIs might induce neuroplastic changes in the structure and function of brain regions involved in regulation of attention, emotion and self-awareness both in long and short term meditators [8]. MBI may also favour emotion regulation by ameliorating the ability of cognitive reappraisal [9], as well as reducing negative dimensions of psychological distress by alleviating anxiety, depression and pain. Mindfulness-based practice has been proved effective also in reducing ruminative thinking both in healthy and depressed individuals [10], thus improving the mental health component of health-related quality of life [11,12,13]. The main results apply mostly to experienced meditators and suggest that the beneficial effects of mindfulness on mental health are mediated by emotion regulation, body awareness and a less static perspective of the self [14]; more recent findings support the hypothesis that similar mechanisms also apply to the general population [15].

Along the years, several MBPs, noteworthy the mindfulness-based stress reduction (MBSR) [16,17] and the mindfulness-based cognitive therapy (MBCT) [18,19], have become very popular because of their positive results among an array of clinical and non-clinical populations [20,21]. Alongside MBSR and MBCT, there are other MBPs [22,23,24], each of them fitting the target population and context and differing from the others in the structural features, such as the duration of each meditation session and the length of the program. Anyway, despite MBPs were originally designed to be delivered in groups, the role of social and group processes has been often neglected in the literature compared to the attention given to individual outcomes [25].

Given the great spread of MBPs, a multitude of different practices and divergent meanings have been attributed to this term. So, MBP needs a stricter definition to achieve a sustainable development of the field. To this regard using a metaphor, a group of researchers [26] compared the textile manufacture to the ‘fabric’ of MBPs they termed ‘warp’ the essential, constant and integral threads that defines an MBP regardless of population or context which make it a MBP; whilst they called ‘weft’ those specific features that characterise each adapted MBP to better fit a particular population or context.

Whilst many studies had been performed in clinical settings, more scarce evidence exists regarding non-clinical settings and general populations. Contextual and social aspects are rarely included as part of MBI. Taking in account these aspects, as well as the characteristic of the target populations, it is possible to develop a tailored intervention.

We developed a program called integral meditation (IM) [27] by taking the ‘wrap’ elements as reference points that represent the central part of our social intervention. The IM, which incorporates mindfulness and aspects from different traditional meditation techniques, is suitable for beginners, since it allows to reach psychophysical well-being fairly quickly. The ‘weft’ features of our IM intervention, its structure and the measured outcomes were adapted according to the study aims and settings and are reported in detail in the Supplementary File S1.

More specifically, the social aspect of our intervention is meditating in a large group of individuals. We hereafter refer to our intervention as social integral meditation (SIM).

Finally, the aim of the current study is to investigate our a priori hypothesis of the causal beneficial effects of our newly developed social intervention (SIM) in meditation beginners from the general population on psychological indicators measured by eight self-report questionnaires encompassing different domains of well-being, mindfulness and emotional functioning.

## 2. Materials and Methods

### 2.1. Participants

Participants were recruited through digital and non-digital advertising. The participation was voluntary based, meaning that most people who joined the study were probably inclined and interested towards meditation. As regard to the exclusion criteria, the subjects should not currently experience severe periods of anxiety or depression, severe mental illness (e.g., hypomania or psychotic episode), recent bereavement or major loss, or any other serious mental or physical health problem that would affect their ability to engage with the course. Additional data from 150 subjects, called population control group (PCG), who was not taking part in the study were also collected, this is to investigate whether any differences exist between people who volunteered to actively participate to the study with people from the general population.

For the sake of space, we discuss the characteristic of the PCG and the differences with the experimental sample in the Supplementary File S1; while study sample characteristics are provided in Table 1.

The sample size was determined by the feasibility of recruitment. For the recruited sample size and for an expected small effect size d = 0.20, power analysis was determined post-hoc using mpe R package and was equal to 0.90 with an alpha equal to 0.006 for at least one endpoint.

### 2.2. Recruitment Process

**Figure 1:**
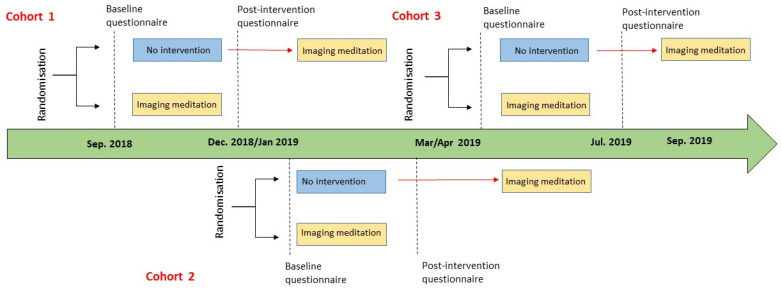
Participant timeline.

Figure 1 graphically depicted the recruitment process: there were three cohorts over a period of 10 months, from September 2018 to June 2019 each with its own participation call. Cohort 1 started the intervention at the end of September 2018 and completed by December 2018; cohort 2 started the intervention in January 2019 and completed it by March 2019; and cohort 3 started the intervention in April 2018 and completed it by July 2019.

After registration, each participant was randomly assigned (1:1) to one group (treated vs. control). All the participants were informed of their group allocation via email and they were invited to complete the questionnaires and to attend the training sessions at specific dates. Because of the nature of the intervention, participants were aware of group allocation for the duration of the study.

In all cohorts, the subjects randomly assigned to the control group at that specific time did not participate in any training session and were allocated to a waiting list. The passive control group was just asked to fill in the same questionnaires as the treated group. For ethical reasons, these subjects were offered to attend the same SIM at subsequent time and were not asked to fill any more questionnaire.

A leaflet stating the aims, the study design, its logistics, the voluntary participation and anonymity were provided with the informed consent to each participant.

### 2.3. Intervention

The core of our SIM intervention is the IM program which comprises 12 meditation classes, given once a week on Monday and lasting approximately 60 min each (see Supplementary File S1 for a detailed description of the program). This IM program, which efficacy was previously tested in a pilot study [27], simultaneously uses breathing, focusing attention, release of physical tensions, thoughts and feeling sensations through internal senses and imagery. IM eases a quick relaxation and more deeply a physical, energetic and spiritual well-being. The experience has shown that IM is well accepted by both novice and experienced meditators. The meditation classes were given in a wide lecture hall of the University of Pavia and the subjects meditated all together in a large group sitting on chairs. Since classes were attended by many participants at the same time, they socialised before and after the classes. Although this was a group intervention, the work required by each participant was highly personal, nevertheless, feeling as part of a community with the same aim greatly helped to get into the meditation state.

### 2.4. Measures

Each participant filled in eight self-report psychological questionnaires at two different time points: at t_0_ (i.e., before the start of the study) and at t_1_ (i.e., at the end of the study); while PCG completed the questionnaire just once. In addition, baseline characteristics and lifestyle of each participant were collected through a background questionnaire.

The questionnaires investigated different psychological dimensions that, according to the literature, can be improved through meditation.

CORE-OM (Clinical Outcomes in Routine Evaluation-Outcomes Measure) [28] measures the global distress of the subject across different dimensions. It is a self-report questionnaire with good psychometric properties composed by 34 items aimed to measure the global distress of the subject across three dimensions: subjective well-being (4 items), problems/symptoms (12 items), life functioning (12 items). In addition, there are six items on risk to self and others that are not regarded as a scale but more as a clinical flag. Item score ranges from 0 to 4. The full-scale can be read as a global index of distress, and each subscale can be used as an index of distress in its specific dimension. The higher the score the higher the distress. A decrease in the mean score after the intervention indicates a diminished global distress or diminished distress relative to the subscale. The CORE-OM possess good psychometric properties and the same applies to the Italian version of the CORE-OM [29] used in our study.

FFMQ (Five Facet Mindfulness Questionnaire) is a 39-item multidimensional assessment tool designed to measure a person’s level of mindfulness [30]. In particular, it is aimed to measure five interrelated components of mindfulness, which are: (1) observing (3 items), (2) describing (3 items), (3) acting with awareness (3 items), (4) non-judging of inner experiences (3 items), (5) non-reactivity to inner experience (3 items). A higher score in the FFMQ full-scale as well as in its subscales reflects a higher level of mindfulness. The questionnaire has showed good psychometric properties both in the English and Italian version, which also shows a similar factorial structure compared to the original one [31]. A higher score after an intervention reflects an improved level of mindfulness.

SWLS (The Satisfaction With Life Scale) [32] is a brief questionnaire used to assess satisfaction with people’s lives as a whole. The scale does not assess satisfaction in specific life domains but measures the global cognitive judgments of one’s life satisfaction. A 5-item scale designed to measure global cognitive judgments of one’s life satisfaction (not a measure of either positive or negative affect). Participants indicate how much they agree or disagree with each of the 5 items using a 7-point scale that ranges from 7 strongly agree to 1 strongly disagree. Higher scores indicate more satisfaction about one’s own life. We used the Italian version of the SWLS, showing a good reliability and concurrent validity [33].

PANAS (Positive Affect Negative Affect Scale) is a 20-items questionnaire that measures two general dimensions [34]: (i) positive affect (PA) reflects the level to which a person feels active, enthusiastic and alert. High PA is a state of high energy, concentration and experiencing pleasure, whereas low PA is characterised by sadness and lethargy. (ii) Negative affect (NA) is a state of general distress and unpleasurable engagement, with low NA reflecting calmness and serenity. This questionnaire has good psychometric properties and the Italian version [35] has been reported as a reliable and valid self-report measure.

PPS (Perceived Stress Scale) [36] measures the perception of stress and the degree to which situations in one’s life are appraised as stressful. The 10 items in the PSS ask about feelings and thoughts during the last month. In each case, respondents are asked how often they felt in a certain way and the answer is given on a 5-point scale. Items were designed to tap how unpredictable, uncontrollable and overloaded respondents find their lives. Higher scores are associated with a greater stress perception. The PSS has good psychometric properties either in English and Italian versions [37].

SCS (Self-compassion scale) measures the thoughts, emotions and behaviours associated with the various components of self-compassion that simply represents compassion turned inward [38]. The questionnaire is made by 26 items that measure how often people respond to feelings of inadequacy or suffering with self-kindness, self-judgment, common humanity, isolation, mindfulness and over-identification. Responses are given on a 5-point scale from ‘almost never’ to ‘almost always’. Higher scores indicate more self-compassion. The SCS has good psychometric properties in the English version and similar ones in the Italian version that we have used [39].

WEMWBS (Warwick-Edinburgh Mental Wellbeing Scale) [40] is a scale of mental well-being including subjective well-being and psychological functioning, in which all items are worded positively and address aspects of positive mental health. The 14 items of this scale measure the frequency of the subject’s attitudes in a 5-point scale from ‘never’ to ‘always’. Higher scores indicate mental well-being. This questionnaire has good psychometric properties valid also in its Italian version [41].

SHS (Subjective Happiness Scale) is a brief questionnaire that requires respondents to make a judgement about how happy they are in order to measure the global subjective happiness [42]. The questionnaire is composed of four items that are rated in a range from 1 to 7. The SHS is based on the subjectivist approach, which considers the respondents’ unique perspective about their own happiness [42]. Using their own happiness criteria, individuals are asked to make an overall judgement about how happy (or unhappy) they are. Higher scores reflect higher levels of subjective happiness. The SHS has good psychometric properties either in the original and the Italian version here used [43].

### 2.5. Data Analysis

This is a two-groups pre-post experimental design. For each cohort, data were collected at two time points: at t_0_, i.e., one week before the start of the program, and at t_1_, i.e., few days after the last meditation class. We did not collect data immediately after the last class to avoid confounding of immediate (but short lasting) effects.

Data were analysed according to intention to treat (IIT). Subjects were excluded from the analysis if they did not fill in all the questionnaires. Questionnaires were scored following the provided guidelines and internal consistency was assessed via Cronbach’s α coefficients [44]. Data were presented as mean ± standard deviation (SD) for continuous variables and frequency distribution for categorical variables. Differences between the two groups (treated vs. controls) at baseline characteristics were investigated using z-tests for continuous variable, chi-squared and Fisher’s exact tests for categorical ones.

Linear mixed model effects (LME) [45] have been applied to evaluate the pre-post treatment changes on each outcome. A nested random intercept for subjects within cohort in the form of 1|cohort/subject had been used to adjust the models for intra-subject variability produced by the two repeated measurements at t_0_ and t_1_ carried out on the same patients, and for potential random variation arising from the way subjects were clustered into cohort. The coefficient of the interaction time*group measures the difference in slopes between the two groups and estimates the effect of the treatment on the outcome, indicating how much more the treatment group is improving over time with respect to the investigated endpoints, compared to control group over the same period. Models were adjusted for sex, age and previous meditation experience. A linear model of the number of classes attended by each participant on each post-pre difference in questionnaires score (Δ) was fitted for testing the possible dose-response effect. Bonferroni adjustment was performed to correct for multiple testing, more precisely eight tests (the number of questionnaires) *p*-value < 0.006 on a 2-sided test were considered as statistically significant. Analysis was performed using R 3.5.1 statistical software [46].

## 3. Results

**Figure 2:**
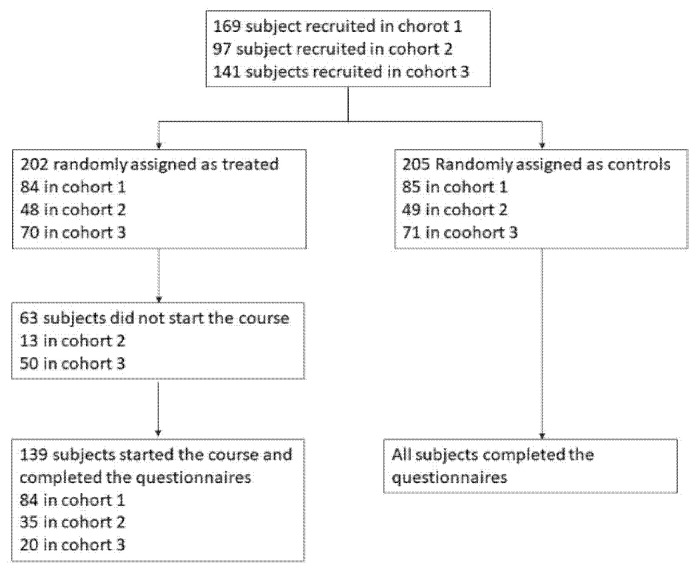
Study profile.

Figure 2 displays the study profile: for the cohort 1 we collected complete pre-post data for 169 subjects (84 treated and 85 controls), in the cohort 2 for 84 subjects (35 treated and 49 controls), and in cohort 3 for 91 subjects (20 treated and 71 controls). It should be noted that some people belonging to the treated group in cohort 2 and 3 left the study after the first few meditation classes or even before starting because of logistic and/or personal reasons, thus not filling the before/after questionnaires. In summary, a total sample of 344 participants (46 male and 298 female) with mean age ± standard deviation (SD) of 41.97 ± (13.71) and range 19–77 were eligible for data analysis. Out of 344 subjects 205 belong to control group while 139 to the treated group. No statistically significant differences were observed between the two groups at baseline characteristics except for ‘favourite music genre’ variable (*p* = 0.01). Results of the analysis of PCG are reported in Supplementary File S1.

In Table 2 mean, SD and internal consistency for each questionnaire both at t_0_ and t_1_ are reported separately in the control and in the treated group.

Table 3 reports for each questionnaire and subscales β coefficient of time*treatment interaction and the corresponding 95% confidence interval (CI), and *p*-value. A statistically significant time*group interaction means that the change in score over time differs between the two groups. Regarding CORE-OM, we found a significant negative interaction for the whole scale (β = −0.20, *p* = 0.0002), and for subjective well-being (β = −0.37, *p* < 0.0001), life functioning (β = −0.20, *p* = 0.0003) and also for symptoms/problems (β = −0.23, *p* = 0.01), this latter did not reach the critical significant threshold after multiple testing adjustment. Negative interaction for CORE-OM and positive interaction for all the remaining scales mean that treatment is beneficial. The time*treatment interaction coefficient was significant and positive for the whole scale of FFMQ (β = 0.20, *p* < 0.0001) and for the subscales observing (β = 0.27, *p* < 0.0001) describing (β = 0.22, *p* < 0.0001), and non-reactivity to inner experience (β = 0.30, *p* < 0.0001) while was not significant in the subscales acting with awareness (β = 0.11, *p* = 0.08) and non-judging of inner experience (β = 0.10, *p* = 0.16).

With regard to SWLS we found a statistically significant positive interaction (β = 1.43, *p* = 0.006), as well as in WEMWBS (β = 4.38, *p* < 0.0001) and in SHS (β = 0.29, *p* = 0.003) questionnaires. In both the two subscales of PANAS we found a statistically significant time*treatment interaction: positive (β = 1.99, *p* = 0.0002) for the PA and negative (β = −1.67, *p* = 0.009) for the NA subscale. In PSS the interaction was significative and negative (β = −2.98, *p* < 0.0001). In SCS we did not find any statistically significant effect neither for the overall scale (β = 0.10, *p* = 0.06) nor for the subscales, except for the subscale mindfulness (β = 0.17, *p* = 0.03) that did not remain statistically significant after multiple testing adjustment.

Among the 139 treated subjects, 80 (58%) attended more than seven meditation classes, 52 (37%) attended a number of classes between three and seven, while 7 (4%) attended less than 2 classes. To test the effect of the number of attended sessions we fitted a linear model in which the dependent variable was the post-pre difference of each questionnaire and the explanatory variable was number of meditation sessions attended (in days). In Table 4 we reported the β coefficient of days with its 95% C.I. and *p*-value. As for the CORE-OM, the number of classes had a statistically significant effect on the overall scale (β = −0.05, *p* = 0.0002), on life functioning (β = −0.06, *p* < 0.0001) and on symptoms/problems (β = −0.02, *p* = 0.0003) subscales while for the other subscales the effect was not significant. A significant effect of number of meditations was also found for the whole scale of FFMQ (β = 0.03, *p* = 0.002), and for the subscale non-judging of inner experience (β = −0.06, *p* = 0.001), while in the other subscales the effect was not significant. The number of meditation sessions had a significant effect also on SWLS questionnaire (β = 0.54, *p* = 0.0004); instead its effect was not significant for PANAS, SHS, PSS, WEMWBS and for SCS except for self-judgment subscale (β = 0.06, *p* = 0.005).

## 4. Discussion

The aim of our study was to investigate if our SIM intervention causally improved psychological factors in the randomly assigned treated compared to the control group.

Our intervention was delivered to a sample from Italian population, where meditation is not so popular and few studies have investigated the effect of meditation-based interventions among the general population. The IM entailed in our intervention can be considered as belonging to the MBP, and it constitutes an example of developing and application of an original MBP constructed by taking the core elements of a MBP [26] and tailoring the variable features according to the characteristics of our study group i.e., adult individuals from the general population and mostly beginners of meditation. Since we have also included the social setting as a part of the intervention, we avoided possible inflations in assessing the intervention effects. As reported by Imel et al. [47], group dynamics can influence the outcomes in intervention such as the MBRS; if group dynamics are not taken in account when comparing a group-based treatment with no treatment condition, this can lead to overestimating the treatment effects.

We observed, a general statistically significant change after the intervention in several of the investigated outcomes in the intervention group as compared to the control one. In particular, after multiple testing adjustment, we observed a statistically significant decreasing score over time in CORE-OM scale as a whole and in the life functioning, and in the subjective well-being subscales, which translates into major beneficial effects. No statistical significance was observed in risk to self and other subscale probably because this refers to the aspects of clinical relevance while our intervention was on healthy subjects. A statistically significant increase in the scores, thus reflecting an improvement of some aspects of mindfulness, was observed in the FFMQ scale and its subscales except for acting with awareness and non-judging of inner experience. A statistically significant improvement in satisfaction of life as measured by SWLS was also observed, as well as in both NA and PA PANAS, thus indicating a better state of energy and enthusiasm and a lower general distress. The latter result is also confirmed in the improvement of PSS, which indicates a reduction of stress perception. On the contrary, the intervention did not affect SCS, except for its mindfulness subscale where a statistically significant improvement was observed but it did not remain significant after multiple testing adjustments. It should be said that SCS has been used in many meditation studies, doubts were casted about its factorial structure [48,49]. Notably, the treated group’s baseline SCS scores are comparable to or higher than those found in two SCS validation studies among Italian samples [39,50], with the exception of the mindfulness subscale. Our interpretation is that our treated group enjoyed a good level of self-compassion even before the treatment, and hence significant changes are less likely to be observed. On the other hand, mindfulness level, which was lower before the treatment, increased.

As to WEMBS, the differences found after treatment indicate a better psychological well-being and a higher happiness as to SHS. Lastly, we found a significant effect of the number of attended meditation classes on the overall score of both CORE-OM and FFMQ, and on SWLS; these results are helpful to understand that for some outcomes we observed a dose–response relationship with the number of meditation classes while for others we did not.

Thus, these results provide evidence of a causal beneficial effect of our intervention on various aspects of the construct of the psychological well-being, both in increasing positive aspects, as mindfulness and subjective happiness, and in reducing negative aspects as distress. As to neuroscience of meditation, it is increasingly recognised that meditation trainings may specifically modulate the cerebral activity of complex brain network including regions such as the prefrontal cortex and the limbic system [51]. These regions play a pivotal role in several cognitive (e.g., attention and memory) and emotional functions (e.g., emotion regulation). Thus, one might speculate that observed multiple beneficial behavioural effects would arise from the modulation exerted by meditation on the brain. This would regulate several aspects of the human well-being-oriented behaviour. Future neuroimaging studies are needed to investigate this hypothesis.

Our study suffers from some limitations: (a) the exact amount of meditation during the study period was not quantifiable since time spent for individual meditation was not available. On the other hand, meditation is thought to be implemented in daily life routine to exploit its benefits, and not just intended to be a treatment that needs a specific amount of time and strict rules to follow. (b) our enrolment was voluntary based, thus not allowing us to extend the inference of its beneficial effect to random individuals from general population but it applies only to individuals who decided to meditate. It should be noted that in order to benefit from a training like ours, participants should be highly motivated to meditate, hence voluntary-based enrolment cannot be avoided. Furthermore, since it is not possible to force an individual to meditate, it is not feasible to randomise a sample from the general population to the treated/control group. (c) There are general issues related to reliability of self-report measures that could have introduced biases in our results [52]; (d) lastly, the length of the questionnaires could have induced a respondent fatigue [53].

## 5. Conclusions

In conclusion, our free and easily accessible SIM, based on an easy-to-learn technique, has produced quick relevant and observable behavioural changes in the participants even after a short training. Meditating in a big group and the social environment were important components of the intervention, allowing us to offer it to as many people as possible and helping the participants to maintain a high level of engagement and motivation throughout the program. It is plausible that our program gathered people who did not have clinically relevant psychological problems and so do not feel the need or the urge to undergo a psychological intervention, but those who were going through a period of particular distress or negative emotional state. It could be possible that our program has supported them to recover to a more balanced psychological state. It is important to underline that our study is not aimed at evaluating the effect of our particular meditation training but of the social intervention including IM, i.e., SIM.

From a public health perspective, as with other behavioural interventions, public health impact depends on scalability and sustainability, both at individual and organisational level. At the individual level, future research might usefully explore the long-term effects of the intervention, investigating if people have continued to meditate and how this affects their wellbeing. At the organisational level, generalisability to other settings, e.g., students, teachers, healthcare practitioners, individuals with work-related stress, and etc., should be further investigated. Thus, our program can be considered as an effective preventive public health intervention to reduce the risk for affective distress and to improve the subject’s general well-being.

## Figures and Tables

**Table 1:** Baseline characteristics of the analysed sample.

Variables	Mean (SD) Controls	Mean (SD) Treated	*p*-Value ^a^
**Age**	41.52 (13.91)	42.64 (13.44)	0.46
	**N (%) controls**	**N (%) treated**	
**Sex**			
Male	29 (8%)	17 (5%)	0.72
Female	176 (51%)	122 (36%)	
**Nationality**			
Italian	195 (57)	136 (39%)	0.31
Non-Italian	10 (3%)	3 (1%)	
**Marital status**			
Cohabitant/married	87 (25%)	56 (16%)	0.37
Unmarried/single	94 (27%)	59 (17%)	
Separated/Divorced	20 (6%)	22 (6%)	
Widowed	3 (1%)	2 (<1%)	
**Number of children**			
0	131 (38%)	80 (23%)	0.29
1	33 (10%)	33 (10%)	
2	37 (11%)	25 (7%)	
≥3	4 (1%)	1 (<1%)	
**Dependent children/family members**			
No	156 (45%)	107 (31%)	0.76
Yes	49 (14%)	30 (9%)	
**Unpaid loans**			
No	164 (48%)	110 (32%)	0.77
Yes	37 (11%)	28 (8%)	
**Education**			
Middle school	8 (2%)	4 (1%)	0.93
High school	80 (23%)	57 (17%)	
Degree	90 (26%)	62 (18%)	
Post-graduate course (e.g., Ph.D.)	27 (%)	16 (%)	
**Job**			
Public or private employee	89 (26%)	69 (20%)	0.67
Freelance (e.g., lawyer, doctor etc.)	30 (9%)	19 (6%)	
Student	50 (14%)	28 (8%)	
Unemployed or looking for a job	9 (3%)	7 (2%)	
Housewife	6 (2%)	4 (1%)	
Retired	14 (4%)	6 (2%)	
Other	6 (2%)	6 (2%)	
**Type of employment agreement**			
Undetermined term	26 (8%)	23 (7%)	0.10
Fixed term	94 (27%)	68 (20%)	
Not applicable	58 (17%)	25 (7%)	
**Employee satisfaction**			
No	60 (18%)	48 (14%)	0.27
Yes	140 (41%)	84 (24%)	
**Sport**			
No	48 (14%)	27 (8%)	0.45
Yes	157 (46%)	112 (33%)	
**Time spent on sport activities**			
Every day	15 (4%)	6 (2%)	0.37
3 times a week	50 (14%)	31 (9%)	
2 times a week	58 (17%)	52 (15%)	
Rarely	44 (13%)	24 (7%)	
Never	37 (11%)	26 (8%)	
**Smoker**			
Yes	30 (9%)	17 (5%)	0.64
No	174 (51%)	121 (35%)	
**Favourite music genre**			
All	45 (13%)	38 (11%)	0.01
No one in particular	31 (9%)	14 (4%)	
Rock	42 (12%)	22 (6%)	
Pop	45 (13%)	23 (7%)	
Jazz	7 (2%)	13 (4%)	
Classic	14 (4%)	21 (6%)	
Other	21 (6%)	7 (2%)	
**Knowledge about meditation**			
Clear idea	75 (22%)	64 (19%)	0.19
Vague idea	105 (30%)	62 (18%)	
Just heard of	23 (7%)	11 (3%)	
Never heard of	2 (1%)	0 (0%)	
**Previous meditation experience**			
Yes	108 (31%)	80 (23%)	0.45
No	97 (28%)	59 (17%)	
**Religious**			
No	114 (33%)	61 (18%)	0.04
Yes	87 (25%)	76 (22%)	
**Number of books read in a year**			
0–1	14 (4%)	8 (2%)	0.92
2–3	51 (15%)	35 (10%)	
>3	139 (40%)	96 (28%)	
**Member of a cultural/sportive association**			
No	136 (40%)	86 (25%)	0.49
Yes	67 (19%)	51 (15%)	
**Diet**			
Mediterranean	173 (50%)	112 (32%)	0.34
Vegetarian	19 (5%)	20 (6%)	
Vegan	5 (1%)	1 (<1%)	
Other	7 (2%)	5 (1%)	
**Use of biological product**			
No	126 (37%)	83 (24%)	0.96
Yes	79 (23%)	54 (16%)	
**Disease/disability**			
No	175 (51%)	118 (34%)	1
Yes	30 (9%)	21 (6%)	
**Addiction**			
No	189 (55%)	127 (37%)	1
Yes	14 (4%)	10 (3%)	
**Have you ever gone to a psychologist**			
No	96 (28%)	73 (21%)	0.35
Yes	109 (32%)	66 (19%)	
**Currently treated by a psychologist**			
No	178 (52%)	116 (34%)	0.57
Yes	27 (8%)	22 (6%)	

^a^*p*-Value for between groups comparison.

**Table 2:** Mean, standard deviation (SD) and internal consistency for each questionnaire and subscale in the two groups (controls and treated) at both time points (t_0_ and t_1_).

Questionnaire	Mean (SD) Controls t_0_	Mean (SD) Treated t_0_	Mean (SD) Controls t_1_	Mean (SD) Treated t_1_	Internal Consistency t_0_	Internal Consistency t_1_
**CORE-OM**						
All Items	1.18 (0.56)	1.07 (0.48)	1.09 (0.57)	0.84 (0.47)	0.93	0.94
Subjective well-being	1.52 (0.90)	1.53 (0.82)	1.55 (0.92)	1.19 (0.77)	0.78	0.81
Life functioning	1.20 (0.56)	1.16 (0.47)	1.19 (0.61)	0.95 (0.50)	0.77	0.84
Symptoms/problems	1.40 (0.76)	1.32 (0.70)	1.31 (0.73)	1.00(0.63)	0.69	0.78
Risk to self and other	0.13 (0.27)	0.09 (0.22)	0.13 (0.31)	0.08 (0.23)	0.89	0.89
**FFMQ**						
All Items	3.32 (0.53)	3.33 (0.52)	3.29 (0.54)	3.50 (0.52)	0.92	0.93
Observing	3.30 (0.66)	3.33 (0.72)	3.24 (0.73)	3.54 (0.75)	0.79	0.83
Describing	3.60 (0.75)	3.51 (0.72)	3.56 (0.79)	3.68 (0.72)	0.91	0.93
Acting with awareness	3.23 (0.77)	3.30 (0.82)	3.22 (0.76)	3.40 (0.74)	0.90	0.90
Non-judging of inner experience	3.49 (0.83)	3.57 (0.78)	3.51 (0.86)	3.69 (0.78)	0.88	0.90
Non reactivity to inner experience	2.90 (0.66)	2.89 (0.67)	2.84 (0.66)	3.13 (0.72)	0.81	0.85
**SWLS**						
All Items	20.35 (7.23)	20.31 (6.72)	20.42 (7.25)	21.73 (6.56)	0.90	0.91
**PANAS**						
Positive	33.45 (6.25)	33.29 (5.57)	32.69 (6.51)	34.55 (5.42)	0.84	0.86
Negative	24.67 (7.66)	23.91 (7.51)	24.34 (7.35)	21.94 (7.10)	0.89	0.89
**PSS**						
All Items	19.17 (7.26)	19.19 (6.94)	18.99 (7.58)	16.01 (6.40)	0.89	0.91
**SCS**						
All Items	3.08 (0.66)	3.12 (0.69)	3.10 (0.68)	3.24 (0.66)	0.95	0.95
Self-kindness	2.77 (0.54)	2.86 (0.55)	2.79 (0.57)	2.92 (0.59)	0.90	0.92
Self-judgment	3.20 (0.88)	3.28 (0.89)	3.25 (0.92)	3.43 (0.90)	0.85	0.88
Common humanity	2.94 (0.89)	2.93 (0.90)	2.97 (0.91)	3.12 (0.83)	0.79	0.80
Isolation	3.54 (0.96)	3.54 (0.94)	3.50 (0.99)	3.65 (0.88)	0.84	0.86
Mindfulness	3.04 (0.86)	2.99 (0.86)	3.00 (0.78)	3.13 (0.82)	0.82	0.80
Over identification	3.05 (0.86)	3.12 (0.85)	3.13 (0.88)	3.22 (0.84)	0.79	0.81
**WEMWBS**						
All Items	48.96 (8.85)	48.48 (7.23)	48.12 (9.06)	51.97 (7.31)	0.91	0.93
**SHS**						
All Items	4.30 (1.30)	4.35 (1.32)	4.25 (1.39)	4.59 (1.21)	0.84	0.86

SD: standard deviation.

**Table 3:** Linear mixed model results. For each questionnaire and subscale β coefficient of time*group interaction with its 95% CI, and *p*-value are reported.

Questionnaire	β Time X Group [95%CI]	*p*-Value
**CORE-OM**		
All Items	−0.20 [−0.30;−0.10]	0.0002 *
Subjective well-being	−0.37 [−0.55;−0.19]	<0.0001 *
Life functioning	−0.20 [−0.31;−0.09]	0.0003 *
Symptoms/problems	−0.23 [−0.37;−0.09]	0.001 *
Risk to self and others	−0.02 [−0.08;0.03]	0.46
**FFMQ**		
All Items	0.20 [0.12;0.28]	<0.0001 *
Observing	0.27 [0.16;0.38]	<0.0001 *
Describing	0.22 [0.11;0.33]	<0.0001 *
Acting with awareness	0.11 [−0.02;0.24]	0.08
Non-judging of inner experience	0.10 [−0.04;0.23]	0.16
Non-reactivity to inner experience	0.30 [0.18;0.43]	<0.0001 *
**SWLS**		
All Items	1.43 [0.42;2.45]	0.006 *
**PANAS**		
Positive	1.99 [0.95;3.04]	0.0002 *
Negative	−1.67 [−2.92;−0.43]	0.009 *
**PSS**		
All Items	−2.98 [−4.25;−1.71]	<0.0001 *
**SCS**		
All Items	0.10 [−0.004;0.21]	0.06
Self-kindness	0.05 [−0.06;0.16]	0.37
Self-judgment	0.11 [−0.06;0.27]	0.20
Common humanity	0.15 [−0.01;0.31]	0.08
Isolation	0.14 [−0.30;0.31]	0.12
Mindfulness	0.17 [0.02;0.33]	0.03
Over identification	0.03 [−0.13;0.18]	0.74
**WEMWBS**		
All Items	4.38 [2.93;5.83]	<0.0001 *
**SHS**		
All Items	0.29 [0.10;0.48]	0.003 *

*p*-Values ≤ 0.006 are considered as statistically significant and are marked with a sign (*). All models are adjusted for sex, age, previous meditation experience and session. The symbol “X” means interaction between two variables.

**Table 4:** Linear model for the effect of the number of meditation sessions attended in the treatment group on each questionnaire and subscale (pre-post difference). β coefficient of days of meditation (β days) with its 95% CI, and *p*-value are reported.

Questionnaire	β Days [95% CI]	*p*-Value
**CORE-OM**		
All Items	−0.05 [−0.08;−0.02]	0.0002 *
Subjective well-being	−0.03 [−0.08; 0.01]	0.173
Life functioning	−0.06 [−0.09;−0.03]	<0.0001 *
Symptoms/problems	−0.02 [−0.10;−0.03]	0.0003 *
Risk to self and others	−0.01 [−0.03;0.004]	0.14
**FFMQ**		
All Items	0.03 [0.01;0.05]	0.002 *
Observing	0.02 [−0.01;0.05]	0.23
Describing	0.02 [−0.002;0.05]	0.07
Acting with awareness	0.04 [0.002;0.8]	0.04
Non-judging of inner experience	0.06 [0.02;0.10]	0.001 *
Non-reactivity to inner experience	0.01 [−0.02;0.04]	0.5
**SWLS**		
All Items	0.54 [0.25;0.84]	0.0004 *
**PANAS**		
Positive	0.05 [−0.21;0.31]	0.72
Negative	−0.31 [−0.67;0.05]	0.09
**PSS**		
All Items	−0.29 [−0.62;0.04]	0.08
**SCS**		
All Items	0.02 [−0.005;0.05]	0.11
Self-kindness	−0.002 [−0.3;0.03]	0.91
Self-judgment	0.06 [0.02;0.10]	0.005 *
Common humanity	0.002 [−0.04;0.05]	0.94
Isolation	0.01 [−0.03;0.06]	0.48
Mindfulness	0.02 [−0.01;0.06]	0.23
Over identification	0.03 [−0.009;0.07]	0.13
**WEMWBS**		
All Items	0.31 [−0.08;0.69]	0.12
**SHS**		
All Items	0.05 [−0.008;0.11]	0.09

*p*-Values ≤ 0.006 are considered as statistically significant and are marked with a sign (*). All models are adjusted for sex, age, previous meditation experience and session.

## Supplementary Materials

The following are available online at https://www.mdpi.com/1660-4601/17/22/8404/s1. File S1: Intervention description and further analysis; File S2: Study raw data.
