# Supplementary material for: Boosting Psychological Well-Being through a Social Mindfulness-Based Intervention in the General Population
Source: Int J Environ Res Public Health. 2020 Nov 13;17(22):8404. doi: 10.3390/ijerph17228404 (PMC7697027; doi:10.3390/ijerph17228404)
Supplement: Supplementary file 1 [file ijerph-17-08404-s001.zip › supelmentary-final/supplementary File S1-final.docx]

Article

**Boosting psychological well-being through a social mindfulness-based intervention in the general population**

**Methods**

Intervention

Our intervention, called *Social Integral Meditation* (SIM), is composed by the original Integral Meditation (IM) training, we developed and tested in a previous pilot study [1], and the social aspects involved in meditating in big groups. Those social aspects refers to: (a) participating in a gathering of people that share the same intention (i.e., to meditate) that may influence the motivation and the feelings that one has toward the activity and (b) to the environmental differences, such as the possibility of socializing, of group meditation compared to individual meditation that may generate psychological effects distinct from those that arise from individual meditation practice.

In the following we first discuss and characterize our meditation training in respect to a classical MBP. The main substantial difference between our intervention approach and MBPs is that MBPs such as MBSR and MBCT have been developed for particular issues such as stress and depression, while ours is aimed to promote psychological well-being and personal growth in a non-clinical population. Nevertheless, there are many common features between MBPs and our intervention which we discuss in here according to the metaphor of “wrap” and “weft” used to represent the fabric of MBP [2].

Our program, we named Integral Meditation (IM) is spiritual in nature and shares the following “wrap” features with MBP:

- it uses mindfulness practices as a vehicle for a systematic training of the mind in the service of developing greater awareness of self and others and it is informed by theories and practice that draw from contemplative traditions, while leaving behind their religious, esoteric and mystical elements;
- it is underpinned by a model of human experience which addresses that causes of human distress and the pathways to relieve it;
- it develops a new relationship with experience characterized by focusing on present moment, and on decentring (i.e., by considering thoughts and feelings as mental events which come and go in the mind as clouds in the sky). The training enables the participants to make a radical shift to their thoughts, feelings and body sensations, as well as to outer circumstances;
- supports the development of greater attentional, emotional and behavioural self-regulation, as well as positive qualities such as compassion, wisdom and equanimity by cultivating an internal climate of friendliness towards experience whether it be pleasant or unpleasant;
- the training develops familiarity with and understanding of the mind and body and appreciation that attention can be regulated, fine-tuned, and optimized through training. Rather like physical training, the training progresses developmentally and sequentially throughout the program.

The “weft” feature of our program resides in:

1. using the imagery to power the concentration and to change the brain waves from beta to alpha/theta leading to a different state of consciousness;
2. using of Tibetan Bowls which invoke a deep state of relaxation which naturally assists one in entering into meditation. They are a quintessential aim to meditation and can be found on private altars, and in temples, monasteries and meditation halls through the world. Meditating on the subtle sound of the Tibetan singing bowl tunes one into the universal sound within and without. Each meditation session from phase 1 to phase 4 was accompanied by this sound.
3. being scheduled over 12 weekly guided sessions lasting 60 min each. The 12 sessions are structured into 4 cycles, each cycle comprises three subsequent meditation sessions. Each cycle is focused on a specific ability as reported below:

Cycle 1: aware diaphragmatic breathing, keeping the posture.

Cycle 2: body scan and awareness of body sensations

Cycle 3: emotions and thoughts feeling and releasing

Cycle 4: imagery activity to change the state of consciousness.

Each cycle also comprises the abilities acquired in the preceding cycle/s, as a result there is an evolution across cycles.

The last cycle, i.e., Cycle 4, also comprises all the abilities acquired in the preceding cycles and is hence structured into the following six final phases:

1. keeping **posture** during meditation, focusing on an aware **diaphragmatic breathing,** body **scan** and awareness of sensations to induce body relaxation leading to an inner emotional calm that is tightly linked to the calm of the physical body; (abilities acquired in Cycle 1 and Cycle 2). Duration 10 min
2. **feeling and examination of emotion and thoughts**; (abilities acquired in Cycle 3). Duration 5 min
3. **visualization of images** (e.g., a sphere of light), colours, relaxing landscapes (e.g., a garden). Visualization powers concentration and calms negative and disturbing dominant emotions. This is not yet a meditation state, but a state leading to the true meditation experience; (abilities acquired in Cycle 4). Duration 20 min
4. **a time of silence** to enjoy the new state of consciousness. After phase iv) the person is free of body sensations, emotions and mental processes and ready to expand his/her own consciousness and open fully him/herself with trust to the union with the higher self and the unified universal field, thus entering into a fine state of meditation to enjoy his/her own personal experience; (abilities acquired in Cycle 4). Duration 15 min
5. **reconnection to the feeling** of the body sensations; (abilities acquired in Cycle 4). Duration 5 min
6. **awareness of the new mental and physical** state (well-being, happiness, peacefulness, serenity etc.). (abilities acquired in Cycle 4). Duration 5 min
7. **sharing the experience:** at the end of each meditation class, the trainer asks the participants how they feel to allow them to freely share their feelings and impressions about the meditation experience.
8. **practice at home:** participants were highly encouraged to practice on their own guided by audio-files on recorded meditation lasting 30 min each.

The meditation classes were given in a wide lecture hall of the University of Pavia and the subjects meditated all together sit on chairs. We take the effect of meditate in group as part of our intervention. The classes were attended by several participants at the same time and they have the chance to talk each other and socialize before and after the class.

The meditation trainer has the competences and the experience for teaching meditation, and he used them to facilitate the beginners to acquire the technique and feel some benefits quickly.

Results

Population control group analysis

Additional data from 150 participants, i.e., a population control group (PCG), who were not taking part in the study were also collected, to investigate if differences exist between people who volunteered to actively participate to the study with people from the general population.

These subjects were asked to complete the questionnaire once. The baseline characteristics and lifestyle of PGC are reported in Supplementary Table S1, in terms of mean (SD) and the frequency distribution respectively for numerical and categorical background variables.

**Table S1:** Baseline characteristics of the population control group (PCG).

| **Variables** | ***Mean (SD) PGC*** |
| --- | --- |
| **Age** | *40.07(15.42)* |
|  |  |
|  | ***n (%) PCG*** |
| **Sex** |  |
| Male | *25 (17%)* |
| Female | *125 (83%)* |
| **Nationality** |  |
| Italian | *149 (99%)* |
| Non-Italian | *1 (1%)* |
| **Marital status** |  |
| Cohabitant/married | *72 (48%)* |
| Unmarried/single | *67 (45%)* |
| Separated/Divorced | *10 (7%)* |
| Widowed | *1 (1%)* |
| **Number of children** |  |
| 0 | *93 (62%)* |
| 1 | *20 (13%)* |
| 2 | *30 (20%)* |
| ≥3 | *7 (5%)* |
| **Dependent children/family members** |  |
| No | *108 (72%)* |
| Yes | *42 (28%)* |
| **Unpaid loans** |  |
| No | *81 (54%)* |
| Yes | *12 (8%)* |
| NA | *57 (38%)* |
| **Education** |  |
| Middle school | *4 (3%)* |
| High school | *64 (43%)* |
| Degree | *62 (41%)* |
| Post-graduate course (e.g. PhD) | *20 (13%)* |
| **Job** |  |
| Public or private employee | *81 (54%)* |
| Freelance (e.g., lawyer, doctor etc) | *10 (7%)* |
| Student | *40 (27%)* |
| Unemployed or looking for a job | *3 (2%)* |
| Housewife | *-* |
| Retired | *4 (3%)* |
| Other | *12 (8%)* |
| **Type of employment agreement** |  |
| Undetermined term | *75 (50%)* |
| Fixed term | *26 (17%)* |
| Not applicable | *49 (33%)* |
| **Employee satisfaction** |  |
| No | *18 (12%)* |
| Yes | *132 (88%)* |
| **Sport** |  |
| No | *55 (37%)* |
| Yes | *95 (63%)* |
| **Time spent on sport activities** |  |
| Every day | *9 (6%)* |
| 3 times a week | *26 (17%)* |
| 2 times a week | *17 (11%)* |
| Rarely | *30 (20%)* |
| Never | *11 (7%)* |
| NA | *57 (38%)* |
| **Smoker** |  |
| Yes | *28 (19%)* |
| No | *122 (81%)* |
| **Favourite music genre** |  |
| All | *28 (17%)* |
| No one in particular | *23 (15%)* |
| Rock | *27 (18%)* |
| Pop | *43 (29%)* |
| Jazz | *6 (4%)* |
| Classic | *11 (7%)* |
| Other | *11 (7%)* |
| **Knowledge about meditation** |  |
| Clear idea | *46 (31%)* |
| Vague idea | *87 (58%)* |
| Just heard | *17 (12%)* |
| Never heard of | *-* |
| **Previous meditation experience** |  |
| Yes | *59 (61%)* |
| No | *91 (39%)* |
| **Religious** |  |
| No | *71 (47%)* |
| Yes | *79 (53%)* |
| **Number of books read in a year** |  |
| 0 -1 | *12 (8%)* |
| 2 -3 | *51 (34%)* |
| >3 | *87 (58%)* |
| **Member of a cultural/sportive association** |  |
| No | *109 (73%)* |
| Yes | *41 (27%)* |
| **Diet** |  |
| Mediterranean | *140 (93%)* |
| Vegetarian | *7 (5%)* |
| Vegan | *1 (1%)* |
| Other | *2 (1%)* |
| **Use of biological product** |  |
| No | *98 (65%)* |
| Yes | *12 (8%)* |
| NA | *40 (38%)* |
| **Disease/disability** |  |
| No | *81(54%)* |
| Yes | *12 (8%)* |
| NA | *57 (38%)* |
| **Addiction** |  |
| No | *80 (53%)* |
| Yes | *13 (9%)* |
| NA | *57 (38%)* |
| **Have you ever gone to a psychologist** |  |
| No | *57 (38%)* |
| Yes | *36 (24%)* |
| NA | *57 (38%)* |
| **Currently treated by a psychologist** |  |
| No | *80 (53%)* |
| Yes | *13 (9%)* |
| *NA* | *57 (38%)* |

Differences between the PCG and our study sample with respect to the baseline characteristics were investigated using z-tests for continuous variable, chi-squared and Fisher’s exact tests for categorical variables. Statistically significant differences were observed between the PCG and our study sample with respect to the following background characteristics: job (*p* = 0.002), type of employment agreement (*p* < 0.0001), employee satisfaction (*p* < 0.0001), sport (*p* = 0.0008), time spent on sport activities (*p* < 0.0001), previous meditation experience (*p* = 0.002), diet (*p* = 0.03), use of biological product (*p* < 0.0001).

In Supplementary Table S2 we reported mean (SD), median (min-max) and internal consistency of psychological questionnaires collected for PCG. To investigate if there were mean differences in the questionnaires scores between our study sample and the PCG both before and after the treatment we performed independent two-samples z-tests, whose results are reported in Supplementary Table S3. Two different analysis were run: in the first we compared the mean of each questionnaire score of all our study sample (control and treated) before the intervention (at t_0_) with the PCG; while in the second, we compared the treated group’s means only after the intervention (at t_1_) with the PCG.

**Table S2.** Mean, standard deviation (SD), median (min-max) and internal consistence for each questionnaire and subscale in the population control group.

| **Questionnaire** | ***n*.** | **Mean (SD)** | **Median (min–max)** | **Internal Consistence** |
| --- | --- | --- | --- | --- |
| **CORE-OM** |  |  |  |  |
| All Items | 150 | 0.965 (0.48) | 0.925(0.09–2.21) | 0.92 |
| Wellbeing | 150 | 1.263 (0.73) | 1.25(0–3) | 0.71 |
| Functioning | 150 | 1.08 (0.50) | 1(0.08–2.58) | 0.77 |
| Symptoms/problems | 150 | 1.17 (0.67) | 1.08(0.08–3) | 0.88 |
| Risk domain | 150 | 0.13 (0.26) | 0(0–1.33) | 0.70 |
| **FFMQ** |  |  |  |  |
| All Items | 150 | 3.40 (0.43) | 3.39(2.51–4.95) | 0.87 |
| Observe | 150 | 3.13 (0.73) | 3.12(1.38–4.88) | 0.80 |
| Describe | 150 | 3.61 (0.69) | - 1. 2–5) | 0.87 |
| Act with awareness | 150 | 3.61 (0.64) | 3.62(2.12–5) | 0.83 |
| Non judge | 150 | 3.68 (0.79) | 3.75(2–5) | 0.87 |
| Non react | 150 | 2.94 (0.67) | 3(1.29–5) | 0.77 |
| **SWLS** |  |  |  |  |
| All Items | 150 | 23.46 (6.23) | 24(8–35) | 0.89 |
| **PANAS** |  |  |  |  |
| Positive | 150 | 34.65 (5.13) | 34(22–49) | 0.82 |
| Negative | 150 | 22.61 (6.50) | 22(10–38) | 0.88 |
| **PSS** |  |  |  |  |
| All Items | 150 | 17.45 (6.45) | 17(3–33) | 0.88 |
| **SCS** |  |  |  |  |
| All Items | 150 | 3.16 (0.75) | 3.17(1.50–4.73) | 0.94 |
| Self-kindness | 150 | 2.64 (0.91) | 2.60(1–5) | 0.88 |
| Self-judgment | 150 | 3.35 (0.98) | 3.40(1–5) | 0.88 |
| Common humanity | 150 | 3.02 (0.85) | 3(1–5) | 0.78 |
| Isolation | 150 | 3.64 (0.99) | 3.75(1–5) | 0.86 |
| Mindfulness | 150 | 3.08 (0.77) | 3(1–5) | 0.76 |
| Over identification | 150 | 3.30 (0.95) | 3.25(1.25–5) | 0.83 |
| **WEMWBS** |  |  |  |  |
| All Items | 150 | 51.19 (6.82) | 52(29–66) | 0.89 |
| **SHS** |  |  |  |  |
| All Items | 150 | 4.59 (1.21) | 4.75(1.50–7) | 0.79 |

**Table S3.** Results of two-sample z test to compare for each questionnaire the mean of (a) our sample study at t_0_ and the population control at t_0_, and (b) our sample study at t1 and the population control at t_0_.

| **Questionnaire** | **z-score pre ^a^** | ***p*-value ^a^** | **95% IC ^a^** | **z-score post ^b^** | ***p*-value ^b^** | **95% IC ^b^** |
| --- | --- | --- | --- | --- | --- | --- |
| ***CORE-OM*** |  |  |  |  |  |  |
| *All Items* | 2.79 | 0.005 | 0.040; 0.231 | −2.20 | 0.028 | −0.232; −0.013 |
| *Wellbeing* | 3.47 | 0.0005 | 0.115; 0.412 | −0.84 | 0.401 | −0.248; 0.099 |
| *Functioning* | 2.16 | 0.031 | 0.010; 0.204 | −2.12 | 0.034 | −0.239; −0.009 |
| *Symptoms/problems* | 2.88 | 0.004 | 0.103; 0.289 | −2.27 | 0.023 | −0.324; −0.024 |
| *Risk domain* | −0.60 | 0.546 | −0.065; 0.034 | −1.79 | 0.073 | −0.109; 0.005 |
| ***FFMQ*** |  |  |  |  |  |  |
| *All Items* | −1.74 | 0.082 | −0.167; 0.010 | 1.68 | 0.092 | −0.016; 0.206 |
| *Observe* | 2.68 | 0.0007 | 0.051; 0.327 | 4.81 | <0.0001 | 0.248; 0.589 |
| *Describe* | −0.55 | 0.580 | −0.164; 0.092 | 1.04 | 0.298 | −0.073; 0.240 |
| *Act with awareness* | −5.25 | <0.0001 | −0.487; −0.222 | −2.54 | 0.011 | −0.367; −0.047 |
| *Non judge* | −1.97 | 0.049 | −0.306; −0.001 | 0.18 | 0.855 | −0.164; 0.198 |
| *Non react* | −0.69 | 0.487 | −0.174; 0.08 | 2.24 | 0.025 | 0.023; 0.344 |
| ***SWLS*** |  |  |  |  |  |  |
| *All Items* | −4.93 | <0.0001 | −4.369; −1.882 | −2.29 | 0.022 | −3.204; −0.248 |
| ***PANAS*** |  |  |  |  |  |  |
| *Positive* | −2.40 | 0.016 | −2.301; −0.231 | −0.16 | 0.873 | −1.318; 1.119 |
| *Negative* | 2.61 | 0.009 | 0.436; 3.064 | −0.83 | 0.403 | −2.244; 0.902 |
| ***PSS*** |  |  |  |  |  |  |
| *All Items* | 2.66 | 0.008 | 0.456; 3.011 | −1.89 | 0.058 | −2.914; 0.050 |
| ***SCS*** |  |  |  |  |  |  |
| *All Items* | −0.82 | 0.411 | −0.197; 0.081 | 0.99 | 0.319 | −0.080; 0.245 |
| *Self-kindness* | 2.10 | 0.035 | 0.011; 0.324 | 3.16 | 0.001 | 0.107; 0.457 |
| *Self-judgment* | −1.24 | 0v226 | −0.298; 0.067 | 0.78 | 0.433 | −0.130; 0.303 |
| *Common humanity* | −0.92 | 0.357 | −0.244; 0.088 | 0.99 | 0.319 | −0.095; 0.292 |
| *Isolation* | −1.05 | 0.295 | −0.289; 0.088 | 0.01 | 0.995 | −0.216; 0.217 |
| *Mindfulness* | −0.61 | 0.543 | −0.201; 0.106 | 0.56 | 0.573 | −0.131; 0.236 |
| *Over identification* | −2.41 | 0.016 | −0.396; −0.041 | −0.73 | 0.466 | 0.284; 0.130 |
| ***WEMWBS*** |  |  |  |  |  |  |
| *All Items* | −3.41 | 0.0007 | −3.822; −1.031 | 0.93 | 0.351 | −0.855; 2.411 |
| ***SHS*** |  |  |  |  |  |  |
| *All Items* | −2.21 | 0.027 | −0.506; −0.030 | 0.06 | 0.952 | −0.271; 0.288 |

Results of this analysis showed that, as regard to whole scale CORE-OM our study sample at t_0_ had higher mean score than PCG (*p* < 0.0001). This difference was also seen in all the questionnaire subscales except for the *risk to self and others* domain (*p* = 0.02) where our study sample had lower score. As for the comparison between the treated group at t_1_ and the PCG the mean difference between the two group of the whole scale CORE-OM appear to be statistically significant (*p* < 0.0001) but the treated group mean scores were lower than PCG. This tendency was observed for all the CORE-OM subscale except of *subjective well-being* (*p* = 0.26). In the FFMQ we found statistically significant differences between our sample at t_0_ and the PCG only in some subscales: in *observing* our sample had an higher score then the control population (*p* = 0.0002), while in *acting with awareness* (*p* < 0.0001) and *non judging of inner experience* (*p* < 0.0001) they had a lower score (*p* = 0.01). After the intervention, our treated group had a statistically significant higher score than the PCG in the FFMQ overall score (*p* = 0.0006), and in *non reactivity to inner experience* subscale, while in *acting with awareness* they had a statistically significant lower score (*p* = 0.0002). In the SWLS, PANAS, WEMWBS, SHS and PSS questionnaires the differences between the two samples were not statistically significant neither at t_0_ nor at t_1_. In SCS questionnaire the only statistically significant difference in mean scores between the two groups was found in the subscale self-*kindness*, in which the experimental samples had higher score than the PCG both at t_0_ (*p* = 0.001) and t_1_ (*p* = 0.0001). Our study sample, which mostly includes beginners of meditation but somehow interested in this kind of techniques, had some differences in the baseline measures compared to a sample recruited from general population. Those differences, despite often non-significant, indicate a trend of lower psychological well-being in the whole experimental sample before the intervention a trend of lower psychological well-being compared to PCG, with exception of some subscales. Those differences were compensated or inverted in sign when we compared the treated group after the intervention with the PCG; the only exceptions were found in the FFMQ subscale *act with awareness* and SWLS which remain significantly lower in the experimental sample even after the intervention. We suppose that, since we are comparing healthy populations, there is a less room for the beneficial effects of short meditation training to be clearly observable in those comparisons. Anyway, it’s plausible that our program gathered those people who didn’t have specific psychological problems, but who were going through a period of higher distress or more negative emotional state, for different reasons and causes.

Being the meditation a technique that is helpful in many conditions, it could be possible that our program have supported those people to establish a more desirable psychological state.

References

1. Fazia, T.; Bubbico, F.; Iliakis, I.; Salvato, G.; Berzuini, G.; Bruno, S.; Bernardinelli, L. Short-Term Meditation Training Fosters Mindfulness and Emotion Regulation: A Pilot Study. *Front. Psychol.* **2020**, *11*, 2828, doi:10.3389/fpsyg.2020.558803.

2. Crane, R.S.; Brewer, J.; Feldman, C.; Kabat-Zinn, J.; Santorelli, S.; Williams, J.M.G.; Kuyken, W. What defines mindfulness-based programs? The warp and the weft. *Psychol. Med.* **2017**, *47*, 990–999, doi:10.1017/S0033291716003317.
